# Co‐expression of the protease furin in *Nicotiana benthamiana* leads to efficient processing of latent transforming growth factor‐β1 into a biologically active protein

**DOI:** 10.1111/pbi.12530

**Published:** 2016-02-02

**Authors:** Ruud H. P. Wilbers, Lotte B. Westerhof, Debbie R. van Raaij, Marloes van Adrichem, Andreas D. Prakasa, Jose L. Lozano‐Torres, Jaap Bakker, Geert Smant, Arjen Schots

**Affiliations:** ^1^ Laboratory of NematologyPlant Sciences DepartmentWageningen University and Research CentreWageningenThe Netherlands

**Keywords:** *Nicotiana benthamiana*, transforming growth factor β1, signal peptide, furin, proteolytic processing, codon optimization

## Abstract

Transforming growth factor beta (TGF‐β) is a signalling molecule that plays a key role in developmental and immunological processes in mammals. Three TGF‐β isoforms exist in humans, and each isoform has unique therapeutic potential. Plants offer a platform for the production of recombinant proteins, which is cheap and easy to scale up and has a low risk of contamination with human pathogens. TGF‐β3 has been produced in plants before using a chloroplast expression system. However, this strategy requires chemical refolding to obtain a biologically active protein. In this study, we investigated the possibility to transiently express active human TGF‐β1 in *Nicotiana benthamiana* plants. We successfully expressed mature TGF‐β1 in the absence of the latency‐associated peptide (LAP) using different strategies, but the obtained proteins were inactive. Upon expression of LAP‐TGF‐β1, we were able to show that processing of the latent complex by a furin‐like protease does not occur *in planta*. The use of a chitinase signal peptide enhanced the expression and secretion of LAP‐TGF‐β1, and co‐expression of human furin enabled the proteolytic processing of latent TGF‐β1. Engineering the plant post‐translational machinery by co‐expressing human furin also enhanced the accumulation of biologically active TGF‐β1. This engineering step is quite remarkable, as furin requires multiple processing steps and correct localization within the secretory pathway to become active. Our data demonstrate that plants can be a suitable platform for the production of complex proteins that rely on specific proteolytic processing.

## Introduction

Transforming growth factor beta (TGF‐β) is a signalling molecule with crucial roles during early development and the regulation of immune responses in mammals. TGF‐β controls cellular processes, including cell proliferation, recognition, differentiation and apoptosis (Blobe *et al*., 2000; Li *et al*., 2006). Three different TGF‐β isoforms (TGF‐β1, TGF‐β2 and TGF‐β3) exist in humans, which are all encoded by a different gene (Govinden and Bhoola, 2003). The TGF‐β genes encode prepro‐TGF‐β that consists of the signal peptide, latency‐associated peptide (LAP) and mature TGF‐β protein. The signal peptide targets the protein to the endoplasmic reticulum (ER) where it is cleaved off. Pro‐TGF‐β forms homodimers and is further processed in the trans‐Golgi by a furin protease. Proteolytic cleavage by this enzyme separates mature TGF‐β from LAP; however, both polypeptides stay noncovalently associated (Dubois *et al*., 1995). This dimeric complex is called the small latency complex (SLC) and keeps mature TGF‐β in an inactive form. Upon secretion of the SLC, LAP binds to latent transforming growth factor binding protein (LTBP) in the extracellular matrix, resulting in a membrane‐bound complex known as the large latency complex (LLC) (Annes *et al*., 2003). TGF‐β is activated by either proteolytic degradation of LAP, reactive oxygen species (ROS), an acidic environment or interaction with integrin receptors (Annes *et al*., 2003). Active TGF‐β is a dimeric protein of ~25 kDa wherein a disulphide bridge connects the two monomers. Each monomer also has 4 internal disulphide bridges that create a cysteine knot structure (Archer *et al*., 1993; Daopin *et al*., 1992; Lin *et al*., 2006; Schlunegger and Grutter, 1992).

The three TGF‐β isoforms share high homology on amino acid level (70–80%, considering mature TGF‐β) and bind to the same receptors (TβRI and TβRII). However, the three isoforms have distinct functions. It is unclear whether these distinct functions are caused by differences in receptor binding affinity or because the isoforms are further regulated by controlled expression in time, place or cell type. Another possibility is that activity can be controlled by different activation mechanisms. For instance, TGF‐β1 and TGF‐β3 can be activated via the interaction with integrin receptors, while TGF‐β2 cannot (Ludbrook *et al*., 2003). TGF‐β1 is best known for its potent immunoregulatory functions, whereas TGF‐β2 and TGF‐β3 play key roles during early embryonic development.

From a pharmaceutical point of view, all three TGF‐β isoforms have potential therapeutic applications. TGF‐β1 maintains immune homeostasis by controlling lymphocyte proliferation, differentiation and survival, but also inhibits the maturation and activation of antigen presenting cells (Geissmann *et al*., 1999; Takeuchi *et al*., 1998). Also, TGF‐β1 plays a key role in the differentiation of regulatory T cells (Tregs), a population of T cells that strongly suppresses immune responses (Chen *et al*., 2003). Thus, TGF‐β1 might be used as a remedy for patients with (chronic) inflammatory diseases, like arthritis, inflammatory bowel disease and multiple sclerosis. However, in the presence of the pro‐inflammatory cytokines IL‐4 or IL‐6, TGF‐β1 gives rise to populations of T helper cells that can promote inflammation (Th9 and Th17 cells, respectively) (Dardalhon *et al*., 2008; Veldhoen *et al*., 2006, 2008). TGF‐β2 also plays a role in controlling the immune system by inducing oral tolerance and suppressing immune responses. Next to that, TGF‐β2 inhibits growth of intestinal epithelial cells and promotes their differentiation. Therefore, TGF‐β2 might be effective in treating inflammatory bowel diseases (Oz *et al*., 2004). A disadvantage of TGF‐β1 and TGF‐β2 is the strong fibrotic response they can elicit. TGF‐β3 on the other hand does not induce fibrosis and therefore has a therapeutic application in scar‐free wound healing (O'Kane and Ferguson, 1997).

For large‐scale production of recombinant TGF‐β, a suitable expression system is required. TGF‐β has been heterologously expressed in bacterial, insect and mammalian expression systems, but expression of TGF‐β in large quantities seems difficult (Zou and Sun, 2004). TGF‐β is improperly folded when using bacterial expression systems and requires chemical refolding. Mammalian expression systems have a low yield and require multistep purification procedures (Zou and Sun, 2006). In the last two decades, plants have emerged as an alternative expression system for the production of recombinant proteins. As eukaryotes, plants are capable of correctly folding complex proteins and perform post‐translational modifications, like N‐glycosylation. Plants are relatively cheap compared with other expression systems and are easy to scale up. One additional advantage for the production of immunoregulatory proteins in plants is that plants do not harbour human pathogens and therefore could be regarded as more safe. Recently, TGF‐β3 has been produced in chloroplasts of *Nicotiana tabacum* (Gisby *et al*., 2011). A synthetic gene was used for chloroplast transformation and yielded up to 3 mg TGF‐β3 per 80 grams of fresh tobacco leaves (37.5 μg/g leaf). However, because TGF‐β3 was expressed in chloroplasts, the protein was not folded correctly due to the many cysteine bridges required for the cysteine knot structure in TGF‐β. Plant‐produced TGF‐β3 therefore had to be chemically refolded upon purification, thereby losing the plants' economical advantage over bacterial expression systems.

In this study, we investigated the possibility to express active TGF‐β1 in *Nicotiana benthamiana* plants without the need for chemical refolding. In a first attempt, we expressed the latent LAP‐TGF‐β1 complex and show that a furin‐like cleavage, which is required for the release of mature TGF‐β1, does not occur *in planta*. We therefore continued employing a strategy wherein mature TGF‐β1 was expressed in the absence of LAP, but unfortunately these proteins lacked activity. Accumulation of latent LAP‐TGF‐β1 was then enhanced to 140 ng/mg total soluble protein (~2.7 μg/g leaf) using a plant chitinase signal peptide and by co‐expressing human furin. Co‐expression of furin also enabled the correct post‐translational processing of LAP‐TGF‐β1 and increased the accumulation of biologically active TGF‐β1 in plant leaves.

## Results

### Furin cleavage does not occur in *Nicotiana benthamiana*

**Figure 1:**
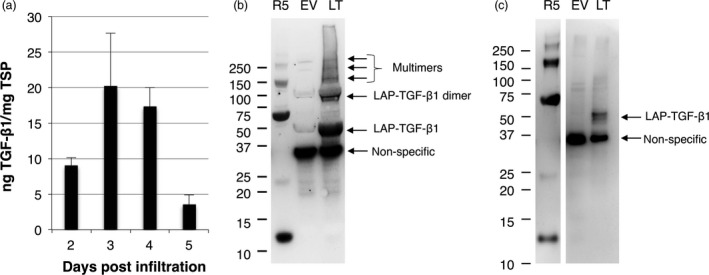
Analysis of LAP‐TGF‐β1 expression in *Nicotiana benthamiana*. Native human LAP‐TGF‐β1 is expressed in *N. benthamiana* leaves, but proteolytic cleavage between the latency‐associated peptide (LAP) and TGF‐β1 by a furin‐like protease does not occur in plants. (a) Yield of human LAP‐TGF‐β1 in crude extracts at 2–5 days postinfiltration (dpi) as determined by ELISA after acid activation (*n *=* *3, error bars indicate standard error). (b/c) Mature TGF‐β‐specific Western blot analysis with a polyclonal antibody under reducing conditions of plant‐produced human LAP‐TGF‐β1 (LT). As controls, empty vector plant extract (EV) or 5 ng recombinant TGF‐β1 (R5) was used. A molecular weight marker indicates protein size in kDa. Extracts were prepared at 3 dpi under neutral pH (b) and at a pH of 3 for acid activation (c).

As LAP could play a role in the folding process of mature TGF‐β, we first expressed the complete open reading frame of human TGF‐β1 in plants. Expression was achieved by agro‐infiltration of leaves of *N. benthamiana* plants, and the yield in crude extracts was determined on 2–5 dpi using a sandwich ELISA (Figure 1a). Crude extracts were analysed with and without acid activation, but this did not influence the detection of TGF‐β1 (only acid‐activated samples are shown in Figure 1a). Maximum yield was found on 3 dpi, reaching 20 ng TGF‐β1/mg TSP.

To investigate the conformation of LAP‐TGF‐β1, Western blot analysis was performed to detect mature TGF‐β1. Leaf extracts were prepared under neutral pH conditions or low pH conditions (pH = 3) to mimic acid activation of the latent TGF‐β1 complex (Figure 1b/c). As expected, recombinant TGF‐β1 from mammalian cells (R5) was detected at ~12.5 kDa, but multimers were detected as well. LAP‐TGF‐β1 migrated at the expected size for latent LAP‐TGF‐β1, just below 50 kDa. When extracted under neutral conditions, also bands at 100, 150 and several bands >150 kDa were detected, which most likely represent dimers and multimers of LAP‐TGF‐β1. These multimers were not observed when LAP‐TGF‐β1 was extracted under acid conditions, which may be due to protein precipitation of large proteins under these conditions. Also a band just below 37 kDa was detected in both blots, but this band was also present in the empty vector (EV) sample. This band is likely to be the result of cross‐reactivity of the polyclonal TGF‐β antibody with a plant protein as this antibody is able to recognize multiple epitopes of TGF‐β1. Our ELISA on the other hand is based on the capture and detection of TGF‐β1 with two different monoclonal antibodies, which makes it very unlikely that our ELISA detects this ~37 kDa plant protein. Also, we have never observed cross‐reactivity with EV control samples. Most importantly, under both neutral and acid conditions mature TGF‐β1 with an expected size of ~12.5 kDa was not detected. This indicates that a furin‐type cleavage between LAP and TGF‐β1 does not occur *in planta*.

### Mature TGF‐β1 can be expressed without LAP, but results in necrosis

**Figure 2:**
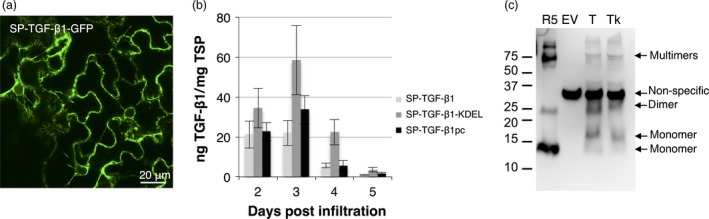
Analysis of mature TGF‐β1 expression in *Nicotiana benthamiana*. (a) Whole mount confocal microscopy output of GFP fused C‐terminally to TGF‐β1 excluding LAP, but including its native signal peptide (SP) at 3 dpi. (b) Yield of SP‐TGF‐β1, SP‐TGF‐β1‐KDEL and plant codon‐optimized SP‐TGF‐β1pc in crude extracts at 2–5 dpi as determined by ELISA (*n *=* *3, error bars indicate standard error). (c) Mature TGF‐β‐specific Western blot analysis with a polyclonal antibody under reducing conditions of plant‐produced SP‐TGF‐β1 (T) and SP‐TGF‐β1‐KDEL (Tk) at 3 dpi. As controls, empty vector plant extract (EV) and 5 ng recombinant TGF‐β1 (R5) were used. A molecular weight marker is indicating size in kDa.

As a furin‐like cleavage did not occur *in planta*, TGF‐β1 was expressed in the absence of the LAP sequence. A construct was created whereby mature TGF‐β1 was fused in frame with its native signal peptide (SP). To investigate whether the signal peptide resulted in adequate uptake into the endoplasmic reticulum (ER), we fused GFP C‐terminally to SP‐TGF‐β1 and followed expression by confocal imaging (Figure 2a). Plants expressing SP‐TGF‐β1‐GFP at 3 dpi showed fluorescence in the nuclear envelope and the endoplasmic reticulum (ER). This indicates that the signal peptide was recognized and that the fusion protein was translocated into the ER.

**Figure 3:**
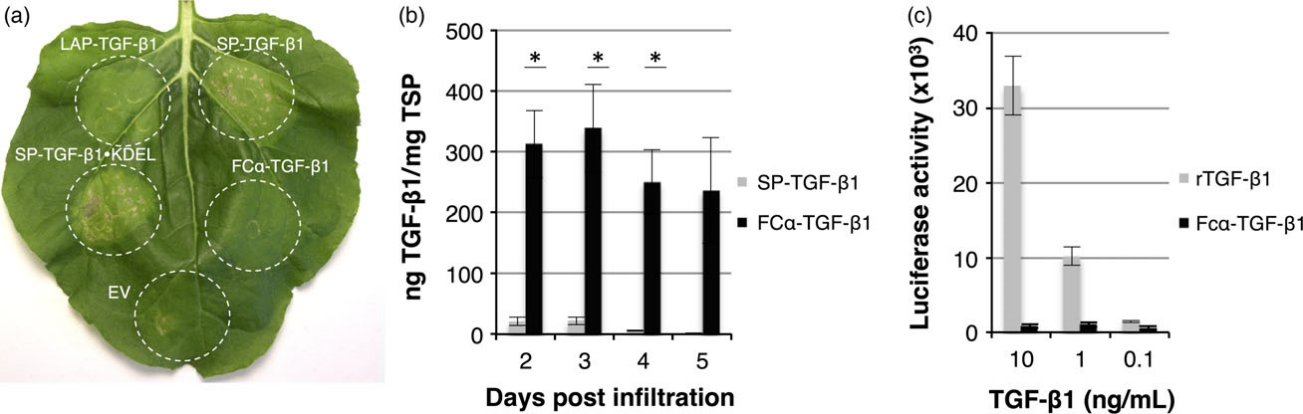
Analysis of the effect of Fcα‐fusion on expression and activity. Necrosis is induced in *Nicotiana benthamiana* leaves upon expression of SP‐TGF‐β1 and SP‐TGF‐β1‐KDEL, but not when fused to LAP or Fcα. (a) Leaf necrosis on 5 days postinfiltration of several TGF‐β1 constructs. (b) Yield of SP‐TGF‐β1 and Fcα‐TGF‐β1 in crude extracts at 2–5 dpi as determined by ELISA (*n *=* *3, error bars indicate standard error). Significant differences as determined by a Welch's t‐test (P < 0.05) between samples are indicated with an asterisk. (c) Biological activity of Fcα‐TGF‐β1 was determined by using MLEC cells containing a TGF‐β1‐inducible luciferase reporter construct. Cells were treated with recombinant TGF‐β1 (rTGF‐β1 from mammalian cells) or plant‐produced Fcα‐TGF‐β1 (from dpi 3), and luciferase expression was measured after overnight incubation.

**Figure 4:**
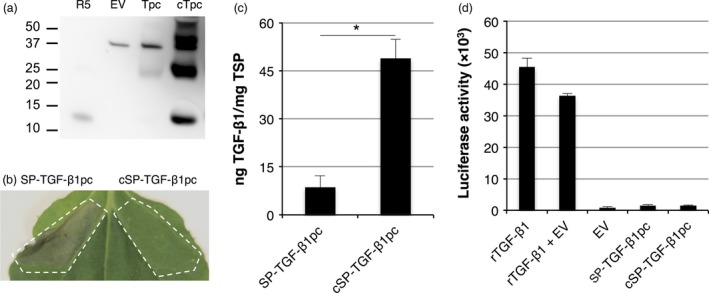
Analysis of the effect of a plant signal peptide on necrosis. Improper cleavage of the native signal peptide results in necrosis. (a) Mature TGF‐β1‐specific Western blot analysis with a polyclonal antibody under reducing conditions of plant‐produced SP‐TGF‐β1pc (Tpc) containing the native signal peptide and cSP‐TGF‐β1pc (cTpc) containing the *Arabidopsis thaliana* chitinase signal peptide at 3 dpi. A molecular weight marker indicates protein size in kDa. As controls, empty vector plant extract (EV) and 5 ng recombinant TGF‐β1 (R5) were used. (b) Leaf necrosis at 5 dpi of SP‐TGF‐β1pc and cSP‐TGF‐β1pc. (c) Yield of SP‐TGF‐β1 and cSP‐TGF‐β1 in crude extracts at 5 dpi as determined by ELISA (*n *=* *3, error bars indicate standard error). Co‐expression of p19 was used in this experiment to further enhance TGF‐β1 expression. (d) Biological activity of 5 ng/mL recombinant TGF‐β1 (rTGF‐β1) and plant‐produced TGF‐β1 (from dpi 5 +  p19) as determined by the induction of luciferase expression in MLEC reporter cells.

Next, we assessed the effect of different strategies to boost the yield of mature TGF‐β1 in plant leaves. Thereto, a C‐terminal KDEL sequence to facilitate ER retention was added or codon use of TGF‐β1 was optimized. The yield of mature TGF‐β1 constructs in crude extracts was determined on 2–5 dpi using a sandwich ELISA (Figure 2b). Although the maximum yield of ER‐retained TGF‐β1 seems slightly higher (up to ~60 ng/mg TSP), no significant differences in maximum yield were observed between mature SP‐TGF‐β1 constructs or with LAP‐TGF‐β1. The yield of SP‐TGF‐β1 quickly drops after 3 dpi, which could be explained by the observation that mature TGF‐β1 induces necrosis in plant leaves. Necrosis appeared as early as 3 dpi for the codon‐optimized SP‐TGF‐β1 and reached its maximum for all constructs at 5 dpi (see Figure 3a and 4b). Necrosis was never observed upon expression of LAP‐TGF‐β1.

When analysing plant extracts by Western blot, several bands were detected for SP‐TGF‐β1 with and without KDEL. The bands of 12.5, 25 and >75 kDa corresponded with the bands detected in the sample with recombinant human TGF‐β1 (Figure 2c) and most likely represent monomers, dimers and multimers of TGF‐β1. However, a band at 16 kDa was also detected and may represent TGF‐β1 with its signal peptide (~3 kDa) still attached. This would mean that although the signal peptide is recognized and enables protein uptake into the ER, it is not cleaved during translocation into the ER.

### Fcα‐fusion of TGF‐β1 enhances yield and prevents necrosis

To increase yield and circumvent improper signal peptide processing, we fused TGF‐β1 to the C‐terminus of a stable partner. The Fc portion of immunoglobulin alpha 1 (Fcα), a natural dimer, was used as a fusion partner allowing dimerization of TGF‐β1. This strategy was previously used to express human interleukin‐10, which is also active as a dimer (Westerhof *et al*., 2012). In contrast to SP‐TGF‐β1 constructs, expression of Fcα‐TGF‐β1 did not result in necrosis in leaves (Figure 3a), even though Fcα‐TGF‐β1 yield increased significantly as analysed in crude extracts at 2–5 dpi (Figure 3b). Fcα‐TGF‐β1 yield was ~16‐fold higher compared with SP‐TGF‐β1, reaching up to 338 ng TGF‐β1/mg TSP on 3 dpi.

Finally, biological activity of Fcα‐TGF‐β1 in crude plant extracts was assessed in a cell‐based assay using mink lung epithelial cells carrying a TGF‐β‐responsive luciferase reporter gene that is activated upon binding to the TGF‐β receptor. Unfortunately, Fcα‐TGF‐β1 was not able to induce luciferase expression in the reporter cell line (Figure 3c). We therefore conclude that Fcα fusion of TGF‐β1 aids in stability of the protein, but the fusion protein lacks activity.

### Improper signal peptide cleavage of TGF‐β1 induces necrosis

To investigate whether an alternative signal peptide would improve signal peptide processing and yield, a construct was created where mature TGF‐β1 was fused in frame with the signal peptide of an *Arabidopsis thaliana* chitinase gene (cSP). The cSP‐TGF‐β1 construct was used for agro‐infiltration and analysed by Western blot (Figure 4a). In extracts of transformed leaves with cSP‐TGF‐β1 (cTpc), a band was detected around ~12.5 kDa, which corresponds with the size of recombinant TGF‐β1 from mammalian cells. A band of ~16 kDa, which resembles TGF‐β1 with an uncleaved SP, was not detected this time. We therefore conclude that the *A. thaliana* chitinase signal peptide is properly cleaved. Besides the band for monomeric TGF‐β1, several other bands representing dimeric and multimeric TGF‐β1 were detected as well, whereas bands for recombinant TGF‐β1 (R5) and SP‐TGF‐β1pc (Tpc) are very faint. Next to that, cSP‐TGF‐β1 does not induce necrosis in *N. benthamiana* leaves from 3 dpi onwards (Figure 4b). We therefore also conclude that the improper processing of the signal peptide was responsible for the necrotic symptoms.

As the cSP‐TGF‐β1 construct does not induce necrosis, we used the silencing inhibitor p19 to further boost TGF‐β1 expression. The yield of cSP‐TGF‐β1 was determined in crude extracts at 5 dpi by sandwich ELISA. Figure 4c reveals that the yield of cSP‐TGF‐β1 was enhanced significantly by p19 when compared to SP‐TGF‐β1 (*P *=* *0.004). This is most likely explained by the lack of necrosis due to the replacement of the signal peptide. However, the maximum yield of ~50 ng TGF‐β1/mg TSP was not significantly higher as native SP‐TGF‐β1‐KDEL at dpi 3 as shown in Figure 2b. With a yield of 50 ng/mg TSP, only 2.5 ng of TGF‐β1 was loaded on gel for Western blot analysis (Figure 4a); however, the band intensity of cSP‐TGF‐β1 is much stronger than the 5 ng recombinant TGF‐β1. Plant‐expressed cSP‐TGF‐β1 could be folded incorrectly, thereby explaining the difference in detection between ELISA and Western blot.

To test whether plant‐produced mature TGF‐β1 is active, crude extracts were applied in the TGF‐β‐responsive luciferase reporter assay (Figure 4d). Recombinant human TGF‐β1 from mammalian cells was able to induce the expression of luciferase in the reporter cells, even in the presence of plant proteins. Unfortunately, mature TGF‐β1 from plants was not active. Thus, replacing the signal peptide of TGF‐β1 can prevent leaf necrosis, but this form of plant‐produced mature TGF‐β1 still lacks biological activity. It is therefore likely that mature TGF‐β1 is not folded correctly in plants and might require LAP for proper folding.

### Proteolytic processing of LAP‐TGF‐β1 in plants requires co‐expression of furin

**Figure 5:**
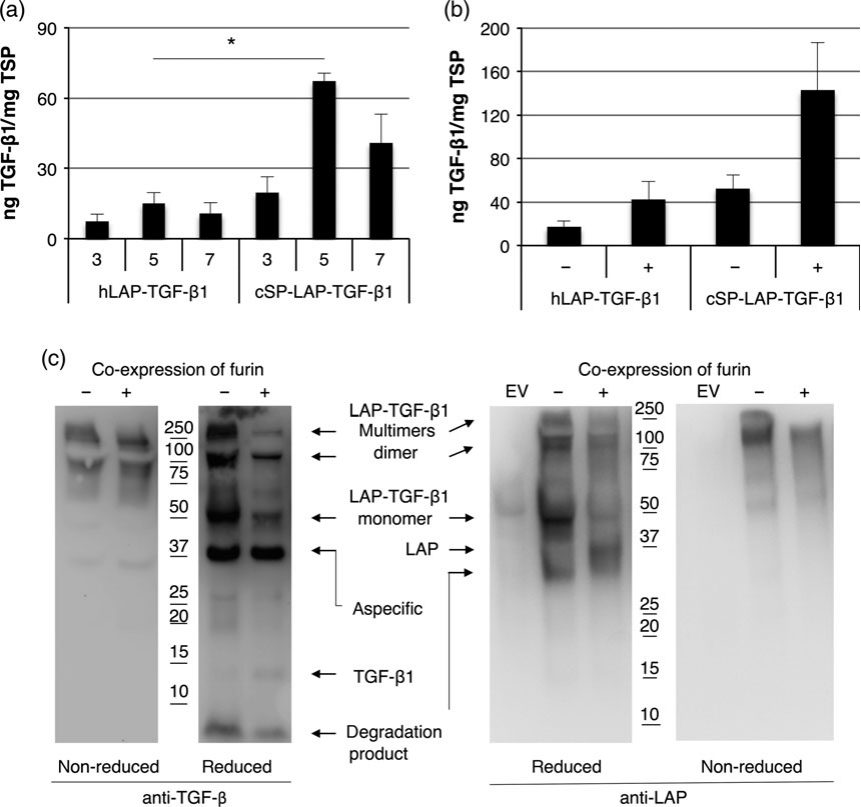
Co‐expression of furin enables proteolytic processing of LAP‐TGF‐β1. (a) Yield of LAP‐TGF‐β1 and cSP‐LAP‐TGF‐β1 in crude extracts at 3, 5 and 7 days postinfiltration (dpi) upon co‐expression of p19 as determined by ELISA after acid activation (*n *=* *3, error bars indicate standard error). Significant differences between samples as determined by a Welch's t‐test (P < 0.05) are indicated with an asterisk. (b) Yield of LAP‐TGF‐β1 constructs in crude extracts at 5 dpi upon co‐expression of furin (*n *=* *3, error bars indicate standard error, +: furin co‐expression). (c) Mature TGF‐β1‐ and LAP‐specific Western blot analysis with polyclonal antibodies under nonreducing and reducing conditions of cSP‐LAP‐TGF‐β1 at dpi 5 (EV: empty vector control).

As mature TGF‐β1 from plants lacks biological activity, we further investigated whether furin is required for the production of TGF‐β1 in plants. We first replaced the native signal peptide of LAP‐TGF‐β1 with the *Arabidopsis* chitinase signal peptide (cSP) and analysed the yield for both LAP‐TGF‐β1 constructs at 3, 5 and 7 dpi while co‐expressing the p19 silencing suppressor. The expression profile for both LAP‐TGF‐β1 constructs is given in Figure 5a. Replacing the native signal peptide with the chitinase signal peptide increased LAP‐TGF‐β1 yield 4.5‐fold (*P *=* *0.002). In a next experiment, we investigated the effect of co‐expression of human furin. Figure 5b shows that co‐expression of furin enhances the yield of both LAP‐TGF‐β1 constructs. Maximum yield of cSP‐LAP‐TGF‐β1 was ~140 ng TGF‐β1/mg TSP, which is 2.7‐fold (*P *=* *0.084) higher than without co‐expression of furin.

Next, we performed a mature TGF‐β1‐specific and LAP‐specific Western blot to analyse the post‐translational processing of LAP‐TGF‐β1 by furin. Under nonreducing conditions, LAP‐TGF‐β1 is detected as a band just above 100 kDa with both antibodies, which is the expected size for the latent LAP‐TGF‐β1 homodimer (Figure 5c; first and fourth panel, respectively). Multimers were detected as well, but no differences were observed upon co‐expression of furin. Under reducing conditions, LAP‐TGF‐β1 was detected as a monomeric band of approximately 50 kDa with the TGF‐β antibody (Figure 5c, second panel). However, the intensity of this band is strongly reduced upon co‐expression of furin. The Western blot probed with anti‐LAP also shows this reduction in intensity for the LAP‐TGF‐β1 monomer (Figure 5c, third panel). Also, a faint band around 12.5 kDa (the size of monomeric TGF‐β1) appears upon co‐expression of furin (Figure 5c, second panel). Furthermore, we detected a band of approximately 37 kDa under reducing conditions when using LAP‐specific antibodies. This likely represents properly processed LAP which should migrate around 37 kDa. However, the anti‐LAP blot also reveals a degradation product of LAP of approximately 30 kDa in both samples. Therefore, LAP‐TGF‐β1 seems to be processed by endogenous proteases in plants as well. Altogether, we conclude that co‐expression of furin enables the correct post‐translational processing of latent LAP‐TGF‐β1.

### Co‐expression of furin enhances the accumulation of active TGF‐β1

**Figure 6:**
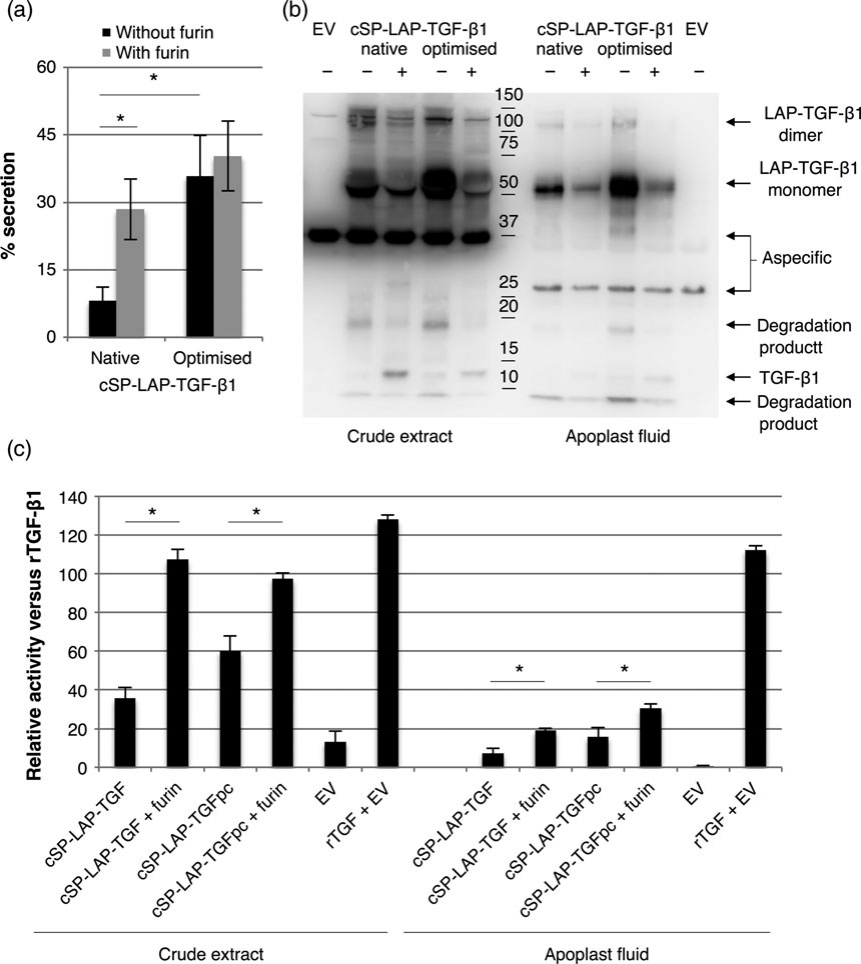
Furin enhances the accumulation of biologically active TGF‐β1. (a) Native and codon‐optimized cSP‐LAP‐TGF‐β1 were transiently expressed in *Nicotiana benthamiana* leaves with or without co‐expression of furin. The percentage of secreted cSP‐LAP‐TGF‐β1 was determined at 5 dpi by determining the amount of cytokine in apoplast fluids versus crude extracts (*n *=* *5, error bars indicate standard error). (cSP:* Arabidopsis thaliana* chitinase signal peptide; TSP: total soluble protein). Asterisk indicates significant differences as determined by a Welch's *t*‐test (*P <* *0.05). (b) Mature TGF‐β1‐specific Western blot analysis with a polyclonal antibody at 5 dpi under reducing conditions of apoplast fluids and crude extracts containing native or codon‐optimized cSP‐LAP‐TGF‐β1 with or without co‐expression of furin (EV: empty vector control; pc: codon‐optimized; +: furin co‐expression). (c) Biological activity of 5 ng/mL recombinant TGF‐β1 (rTGF‐β1) and plant‐produced TGF‐β1 (from dpi 5) as determined by the induction of luciferase expression in MLEC cells. Relative activity of plant‐produced TGF‐β1 versus recombinant TGF‐β1 (in %) is given (*n *=* *3, error bars indicate standard error).

To investigate the efficiency of furin cleavage, we isolated latent TGF‐β1 from the apoplastic space of agro‐infiltrated leaves at 5 dpi and analysed the conformation on Western blot. First, we determined the secretion efficiency of TGF‐β1 into the apoplast. In Figure 6a, we demonstrate that co‐expression of furin significantly increases the secretion efficiency of native cSP‐LAP‐TGF‐β1 (*P *=* *0.018). Quite unexpectedly, secretion efficiency of cSP‐LAP‐TGF‐β1 was also significantly increased upon codon optimization (*P *=* *0.017) and furin did not further increase secretion of codon‐optimized cSP‐LAP‐TGF‐β1.

Next, the efficiency of furin cleavage was evaluated by performing a TGF‐β1‐specific Western blot on both crude extracts and apoplastic fluids (Figure 6b). As described in the previous paragraph, the intensity of the 50 kDa band, which resembles the size of unprocessed LAP‐TGF‐β1, is reduced in both crude extracts and apoplast fluids upon furin co‐expression. However, the processing of LAP‐TGF‐β1 is not complete as unprocessed LAP‐TGF‐β1 is still detected in the apoplast. We also observed clear bands around 12.5 kDa upon furin co‐expression. The intensity of this monomeric mature TGF‐β1 is stronger in crude extracts, which is striking as the absolute amount of TGF‐β1 on gel should be higher for apoplast fluids according to the ELISA. Furthermore, several degradation products were detected, which indicates that LAP‐TGF‐β1 is sensitive to the activity of endogenous proteases.

To evaluate the biological activity of cSP‐LAP‐TGF‐β1 constructs, the effect of acid‐activated crude extracts or apoplast fluids on mink lung epithelial cells carrying the TGF‐β1‐responsive luciferase reporter gene was assessed. Figure 6c shows that co‐expression of furin enhances the biological activity of TGF‐β1 in crude extracts to a similar level as recombinant TGF‐β1. Unfortunately, cSP‐LAP‐TGF‐β1 isolated from the apoplast was reduced in its activity when compared to crude extracts and recombinant TGF‐β1. It is therefore likely that cSP‐LAP‐TGF‐β1 is degraded by endogenous proteases in the apoplast and therefore loses activity.

## Discussion

Here, we show that expression of biologically active TGF‐β without the requirement of chemical refolding is feasible in plants. Previously, TGF‐β3 was successfully produced in chloroplasts of *Nicotiana tabacum* plants (Gisby *et al*., 2011) and yielded considerable amounts of this protein (37.5 μg/g FW and 80% purity). However, this strategy required chemical refolding of purified TGF‐β3 to obtain biological activity. Therefore, chloroplast expression, as a plant‐based expression system, loses one of its major advantages over bacterial expression systems, namely cost‐effectiveness. When we studied the transient expression of the whole open reading frame of the TGF‐β1 gene in *N. benthamiana* plants, the obtained yield of TGF‐β1 was ~20 ng/mg TSP. This yield was considerably lower compared with many other proteins heterologously expressed in plants, like antibodies or other cytokines (Westerhof *et al*., 2012, 2014). More importantly, we revealed that a furin‐like cleavage between LAP and TGF‐β1 does not occur in *N. benthamiana* plants. This furin‐like cleavage is required to release active TGF‐β1 from the latent complex. Furin is a mammalian subtilisin‐/kex2p‐like endoprotease. Although a kex2p‐like pathway was shown to exist in *N. tabacum* (Kinal *et al*., 1995), no furin cleavage of LAP‐TGF‐β1 was observed in our expression system. Hence, it is likely that a protease with the right specificity does not exist in *N. benthamiana*. The lack of furin cleavage might be a crucial factor that limits the yield of TGF‐β1 in plants.

To circumvent the post‐translational processing of LAP‐TGF‐β1, we expressed mature TGF‐β1 as an in‐frame fusion with its native signal peptide. We confirmed that the signal peptide of TGF‐β1 is functional by monitoring localization of a GFP fusion protein with confocal imaging, as SP‐TGF‐β1 is taken up into the secretory pathway. However, after translocation into the ER, the signal peptide is not completely removed from the mature protein. When the signal peptide stays attached to mature TGF‐β1, it could compromise the function of the plant ER. The unprocessed signal peptide could act as membrane anchor (High and Dobberstein, 1992), thereby facilitating accumulation of unprocessed TGF‐β1 in the ER. On the other hand, the signal peptide could also affect the folding of TGF‐β1 and trigger the unfolded protein response. Disturbance of ER function could ultimately lead to necrosis (Ye *et al*., 2011). This disturbance of ER function may explain the necrosis that we observed upon expression of SP‐TGF‐β1.

Replacing the signal peptide with an *A. thaliana* chitinase signal peptide circumvents improper cleavage of the TGF‐β1 signal peptide, prevented the induction of necrosis and increased yield of mature TGF‐β1. Surprisingly, signal peptide replacement with the chitinase signal peptide also significantly increased the yield of LAP‐TGF‐β1. The chitinase signal peptide may also influence expression by changing the mRNA secondary structure at the 5′‐end. The secondary structure of the 5′ UTR sequence containing the *Alfalfa mosaic virus* RNA 4 (AlMV) leader and the first 40 nucleotides of LAP‐TGF‐β1 was predicted by the Vienna RNA fold software (Lorenz *et al*., 2011). In Figure S1, drawings of the minimum free energy predictions for both LAP‐TGF‐β1 genes are given. This prediction reveals that the combination of the AIMV leader with the chitinase signal peptide increases the minimal free folding energy 6.5‐fold, making the secondary structure less stable. Furthermore, strong GC‐rich secondary structures are present downstream of the start codon in the native signal peptide. As low minimal free folding energy has been suggested to enhance translation initiation (Hall *et al*., 1982; Tuller *et al*., 2010), these observed differences could explain differences in yield.

Even though the yield was improved, a major bottleneck for the accumulation of mature TGF‐β1 seems to be low stability or misfolding of the protein. Plants are rich in proteases, and a wide variety of proteases reside within the secretory pathway of plants as well as in the apoplast (Goulet *et al*., 2012; van der Hoorn, 2008). It is therefore inevitable that recombinant secretory proteins encounter proteases on their way out. Proteolytic processing is regarded as one of the largest bottlenecks of plant expression systems. A classic example is the unintended processing of antibodies when expressed in plants (De Muynck *et al*., 2010). A common strategy to increase recombinant protein yield and circumvent proteolytic degradation is to retain the protein in the ER by using a C‐terminal KDEL targeting sequence (Schouten *et al*., 1996). The ER is regarded as a protein friendly environment due to the absence of many proteases. We have used ER retention in order to increase the yield of TGF‐β1, but this strategy did not result in significantly higher yields. As cytokines are inherently unstable, we also attempted to increase the yield of TGF‐β1 by fusing them to a stable partner. In our study, fusion of TGF‐β1 to Fcα increased the yield 16‐fold, but led to the production of inactive TGF‐β1. The observation that plant‐produced mature TGF‐β1 or Fcα‐TGF‐β1 is inactive is therefore most likely explained by misfolding of TGF‐β1 in the absence of LAP. This idea is further supported by the fact that we observed a strong difference in the detection of mature TGF‐β1 by ELISA and Western blot. Misfolded TGF‐β1 is likely not detected properly in a sandwich ELISA, but might be detected by Western blot under reducing conditions as continuous epitopes become available. Proper folding of biologically active TGF‐β1 likely requires expression of the full LAP‐TGF‐β1 gene.

Post‐translational modification of LAP‐TGF‐β1 is a complex process, which is controlled at multiple levels. The secretion of biologically active TGF‐β1 dimers first of all requires the dimerization of the LAP‐TGF‐β1 proprotein, which occurs in the endoplasmic reticulum (Gray and Mason, 1990). LAP‐TGF‐β1 is also N‐glycosylated at three residues (82, 136 and 176) and the presence of the N‐glycans on residues Asn82 and Asn136 was shown to be required for proper secretion of LAP‐TGF‐β1 (Brunner *et al*., 1992; Sha *et al*., 1989). Yet, secretion of latent TGF‐β1 is a slow process as LAP‐TGF‐β1 is retained within the *cis*‐Golgi in an Endo H‐sensitive form (Miyazono *et al*., 1992). *Cis*‐Golgi retention of LAP‐TGF‐β1 is presumed to be caused by its capture by a sequestering protein, such as the chaperone GRP78 (Oida and Weiner, 2010). Retention of LAP‐TGF‐β1 in the ER/cis‐Golgi is the limiting step for secretion and furin processing (Oida and Weiner, 2010). Only properly glycosylated LAP‐TGF‐β1 is transported to the *trans*‐Golgi where it is processed by furin (Dubois *et al*., 1995).

Within our study, we co‐expressed human furin with LAP‐TGF‐β1 to investigate the proper proteolytic processing of LAP and TGF‐β1 in plants. First of all, we observed that co‐expression of furin slightly increased the yield of LAP‐TGF‐β1 by 2.7‐fold (*P *=* *0.084), but significantly enhanced the secretion of cSP‐LAP‐TGF‐β1 into the apoplast (*P *=* *0.002). We also observed that the majority of extracted LAP‐TGF‐β1 is properly processed by co‐expressed furin (Figures 5c and 6b). This engineering strategy is quite remarkable as furin requires multiple autocatalytic processing steps and correct localization within the secretory pathway to become active (Vey *et al*., 1994).

Co‐expression of furin also enhanced the accumulation of biologically active TGF‐β1 in *N. benthamiana* leaves, but activity of secreted LAP‐TGF‐β1 was reduced. This is most likely explained by the fact that LAP‐TGF‐β1 is sensitive to the activity of plant endogenous proteases that reside within the apoplast. A common activation mechanism for latent TGF‐β1 in mammals is the proteolytic degradation of LAP by proteases, like plasmin, matrix metalloproteinases or thrombospondin (Boor *et al*., 2010). It seems that endogenous proteases with similar activity exist in plants and therefore future work has to focus on how the stability of LAP‐TGF‐β1 in the apoplast can be enhanced. When undesired proteolytic processing of LAP‐TGF‐β1 can be prevented, co‐expression of furin will enable the production of active TGF‐β1 in plants without the need for chemical refolding.

Our study demonstrates that the production of biologically active TGF‐β1 in plants is feasible without the need for chemical refolding. Thereby, we avoid difficult and expensive chemical refolding steps, which are required upon bacterial or chloroplast expression. We also show that the post‐translational machinery of plants can be engineered to allow proteolytic processing of heterologous expressed mammalian proteins. Therefore, this study highlights the fact that plants are a suitable expression platform for the production of complex glycoproteins, like TGF‐β1, that rely on correct post‐translational processing for biological activity. This engineering strategy can further facilitate plant‐based expression of other proproteins that rely on furin cleavage, like neural growth factor (NGF), bone‐morphogenic proteins (BMPs) or even the Ebola Zaire envelope glycoprotein (Thomas, 2002). Furthermore, co‐expression of other proteases can be explored as well to facilitate plant‐based expression of a variety of complex proproteins.

## Experimental procedures

### Construct design

The complete native open reading frame (ORF) of human (h)LAP‐TGF‐β1 was amplified from the MegaMan™ Human Transcriptome cDNA library (Bio‐Connect BV, Huissen, The Netherlands). Similarly, mouse LAP‐TGF‐β1 was amplified from the FirstChoice™ PCR‐Ready Mouse Spleen cDNA library (Life Technologies, Bleiswijk, The Netherlands). To remove LAP from the mature ORF, mature hTGF‐β1 was re‐amplified whereby a SacII restriction site was introduced at the 5′ end. This SacII was used to clone mature human TGF‐β1 in frame with the mouse signal peptide, as SacII was uniquely present in the mouse (and not human) LAP‐TGF‐β1 signal peptide. Thereafter, SP‐TGF‐β1 was re‐amplified to introduce the ER retention signal KDEL at the 3′ end of the gene. The complete ORF of human furin (NM_002569) was amplified from the MegaMan™ Human Transcriptome cDNA library. An internal BspHI site was removed by means of overlap‐extension PCR to ensure subsequent cloning into pBIN^+^.

To create a GFP fusion of hTGF‐β1, the enhanced green fluorescent protein (eGFP) gene was re‐amplified and used to replace the mature hIL‐10 sequence in the previously published Fcα‐IL‐10 construct (Westerhof *et al*., 2012) using SpeI/KpnI. Similarly, hTGF‐β1 was re‐amplified and used to replace Fcα in the same Fcα‐hIL‐10 construct using NcoI/SacI. In this way, a SP‐TGF‐β1‐GFP construct was created where a glycine–serine linker separated hTGF‐β1 and GFP. To create a Fcα‐fusion of hTGF‐β1, mature hTGF‐β1 (SpeI/KpnI) was re‐amplified and used to replace the mature hIL‐10 sequence in the previously mentioned Fcα‐IL‐10 construct.

The mature TGF‐β1 sequence was codon‐optimized using an in‐house optimization procedure and ordered at GeneArt (Life Technologies). The codon‐optimized TGF‐β1 gene was cloned in frame with the *Arabidopsis* chitinase signal peptide (cSP, AAM10081.1) by means of overlap‐extension PCR. Similarly, the mature sequence for native LAP‐TGF‐β1 sequence was cloned in frame with cSP.

All construct sequences were confirmed by sequencing (Macrogen, Amsterdam, The Netherlands) in the expression vectors pBIN^+^ and pHYG (Westerhof *et al*., 2012) and an overview of all constructs is given in Figure S2. In pBIN^+^ and pHYG, expression is controlled by the 35S promoter of the *Cauliflower mosaic virus* with duplicated enhancer (d35S) and the *Agrobacterium tumefaciens* nopaline synthase transcription terminator (Tnos). A 5′ leader sequence of the *Alfalfa mosaic virus* RNA 4 (AlMV) is included between the promoter and construct to boost translation. The expression vectors were subsequently transformed to *Agrobacterium tumefaciens* strain MOG101 for plant expression. In some experiments, the silencing suppressor p19 from tomato bushy stunt virus was co‐infiltrated to enhance heterologous expression. The vector pBIN61‐p19 was obtained from Dr. D. Baulcombe (Voinnet *et al*., 2003).

### Agro‐infiltration of *Nicotiana benthamiana*

*Agrobacterium tumefaciens* clones were cultured for 16 h at 28 °C/250 rpm in LB medium (10 g/L pepton 140, 5 g/L yeast extract, 10 g/L NaCl with pH = 7.0) containing 50 μg/mL kanamycin, 20 μg/mL rifampicin and 20 μm acetosyringone. The bacteria were resuspended in MMA infiltration medium (20 g/L sucrose, 5 g/L MS salts, 1.95 g/L MES, pH5.6) containing 200 μm acetosyringone to obtain an OD (600 nm) of 1. For co‐infiltration experiments, a final OD of 0.5 of each individual culture was used. After 1–2 h of incubation at room temperature, the two youngest fully expanded leaves of 5‐ to 6‐week‐old *N. benthamiana* plants were infiltrated completely.

### Total soluble protein extraction

Leaves were immediately snap‐frozen upon harvesting and homogenized in liquid nitrogen. Homogenized plant material was ground in ice‐cold extraction buffer (50 mm phosphate‐buffered saline (PBS) pH = 7.4, 100 mm NaCl, 0.1% v/v Tween‐20 and 2% w/v immobilized polyvinylpolypyrrolidone (PVPP)) using 2 mL/g fresh weight. For extraction at low pH, a 30 mm sodium citrate buffer (30 mm sodium citrate buffer pH = 3, 100 mm NaCl, 0.1% v/v Tween‐20 and 2% w/v PVPP) was used. Crude extracts were clarified by centrifugation at 16 100 *g* for 5 min at 4 °C, and supernatants were directly used in an ELISA and BCA protein assay.

### Isolation of apoplast fluid

Leaves were vacuum‐infiltrated with ice‐cold extraction buffer (50 mm phosphate‐buffered saline (pH = 8), 100 mm NaCl and 0.1% v/v Tween‐20). Leaves were centrifuged for 20 min at 2000 g to isolate apoplast fluid. Apoplast fluids were clarified by centrifugation at 16 000 g at 4 °C. To determine the percentage of secretion, the remaining leaf material was processed as described above.

### Quantification of human TGF‐β1 protein levels

TGF‐β1 protein concentration in crude plant extracts was determined by ELISA. Samples containing LAP‐TGF‐β1 were activated by the addition of 1M hydrochloric acid (10 μL acid/50 μL sample) and subsequently neutralized after 10 min by adding the same volume of 1M sodium hydroxide. The human TGF‐β1 Ready‐SET‐Go!^®^ ELISA kit (eBioscience, Vienna, Austria) were used according to the supplier's protocol. The ELISA is based on the capture of TGF‐β1 with a monoclonal antibody and subsequent detection with a biotinylated monoclonal antibody and shows no cross‐reactivity to plant proteins. For sample comparison, the total soluble protein (TSP) content was determined by the BCA method (Life Technologies) according to the supplier's protocol using bovine serum albumin (BSA) as a standard.

### Protein analysis by Western blot

Soluble plant proteins (~50 μg for crude extract or ~20 μg for apoplast fluid) were separated under reducing or nonreducing conditions by SDS‐PAGE on a 12% Bis‐Tris gel. Recombinant human TGF‐β1 (R&D Systems, Abingdon, UK) was used as a control. Proteins were transferred to a PVDF membrane (Life Technologies) by a wet blotting procedure. Thereafter, the membrane was blocked in PBST‐BL (PBS containing 0.1% v/v Tween‐20 and 5% w/v nonfat dry milk powder) for 1 h at room temperature, followed by overnight incubation with a rabbit anti‐TGF‐β polyclonal antibody (Bioké, Leiden, The Netherlands) or a goat anti‐LAP polyclonal antibody (R&D Systems) in PBST (including 0.1% w/v bovine serum albumin) at 4 °C. HRP‐conjugated secondary antibodies (Jackson ImmunoResearch, Suffolk, UK) were used for visualization. Finally, the SuperSignal West Femto substrate (Pierce) was used to detect the HRP‐conjugated antibodies.

### Confocal microscopy

Plants were agro‐infiltrated with *A. tumefaciens* harbouring the pHYG vector containing the expression cassette encoding N‐terminal fusions of human TGF‐β1 to GFP as described previously. Leaves were taken from the plant at 3 days postinfiltration, and small sections were examined from the abaxial side using a Zeiss LSM510 (Zeiss, Oberkochen, Germany) confocal laser‐scanning microscope in combination with an argon ion laser supplying a 488 nm wavelength.

### Biological activity assay

The mink lung epithelial cell line with TGF‐β‐inducible luciferase reporter construct (Abe *et al*., 1994) was kindly provided by Dr. C. Arancibia (Oxford University). MLEC cells were maintained at 37 °C with 5% CO_2_ in RPMI‐1640 medium containing 4 mm L‐glutamine and 25 mm HEPES and supplemented with 10% foetal calf serum, 50 U/mL penicillin, 50 μg/mL streptomycin and 200 μg/mL G418. MLEC cells were harvested by trypsinization and seeded at a density of 1.5 × 10^6 ^cells/mL in 96‐well plates and were allowed to rest for 4 h in medium without G418 prior to bioassays. For bioassays, cells were treated with 0.1–10 ng/mL plant‐produced TGF‐β1 (acid activated) or recombinant human TGF‐β1 expressed in Chinese hamster ovary cells (R&D Systems). Recombinant TGF‐β1 was supplemented with EV plant extract to keep plant protein levels equal. After overnight incubation, luciferase expression was analysed using the Bright‐Glo^™^ Luciferase assay system (Promega, Leiden, The Netherlands) according to the supplier's protocol. Luminescence was measured using the FLUOstar OPTIMA microplate reader (BMG Labtech, Ortenberg, Germany).

### Data analysis

All data shown in the figures (unless stated otherwise) indicate the average of at least three biological replicates (*n*). In the figure legends, *n* is indicated and error bars indicate standard error. Significant differences between samples were calculated using the Welch's t‐test and regarded as significant when *P *<* *0.05. Significant differences are indicated in the figures by an asterisk (*).

## Supporting information


**Figure S1** Secondary mRNA structure prediction of the 5′ UTR sequence containing the *Alfalfa mosaic virus* RNA 4 (AlMV) leader and the first 40 nucleotides of the LAP‐TGF‐β1 genes by the Vienna RNA fold software.

**Figure S2** A schematic overview of all the constructs used in this study.
