# Supplementary figures and images for: Co‐expression of the protease furin in Nicotiana benthamiana leads to efficient processing of latent transforming growth factor‐β1 into a biologically active protein
Source: Plant Biotechnol J. 2016 Feb 2;14(8):1695–704. doi: 10.1111/pbi.12530 (PMC5067602; doi:10.1111/pbi.12530)

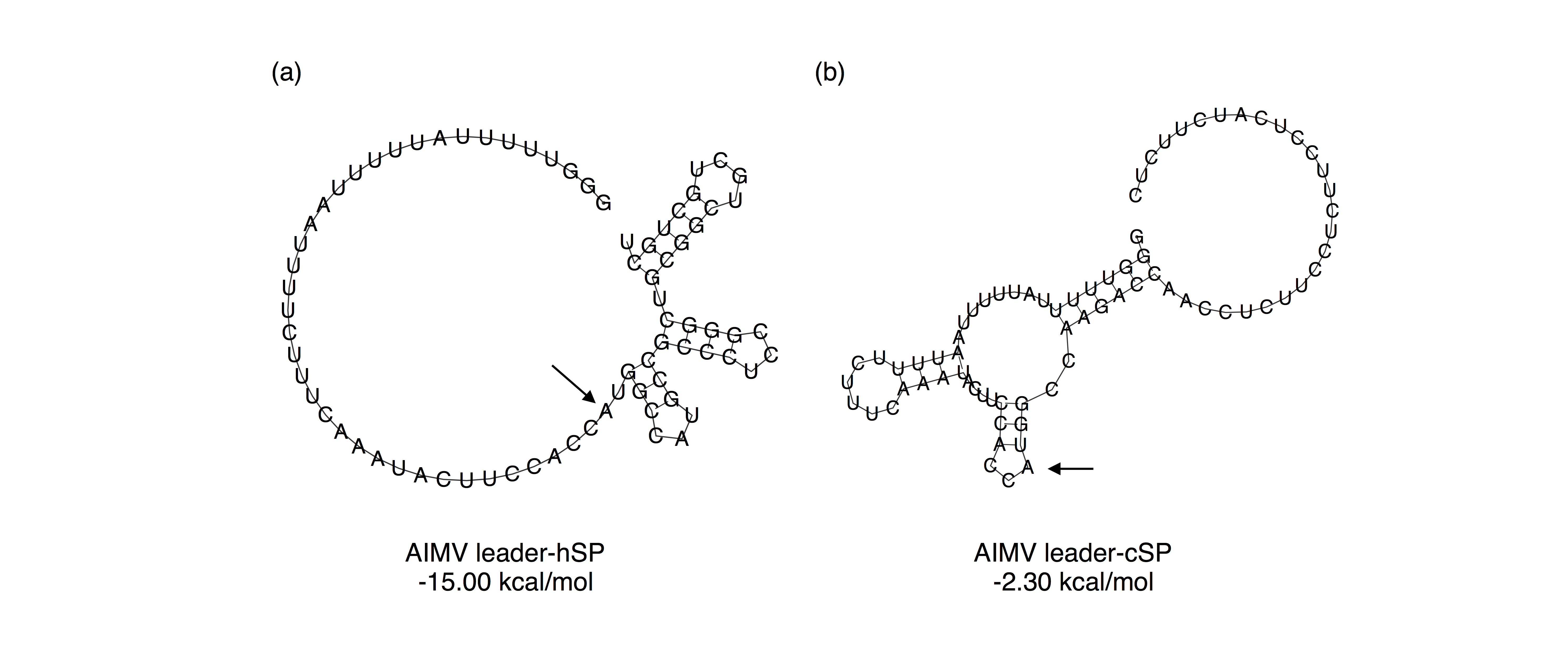

Supplement: Supplementary file 1 — Figure S1 Secondary mRNA structure prediction of the 5′ UTR sequence containing the Alfalfa mosaic virus RNA 4 (AlMV) leader and the first 40 nucleotides of the LAP‐TGF‐β1 genes by the Vienna RNA fold software. [file PBI-14-1695-s001.tiff]

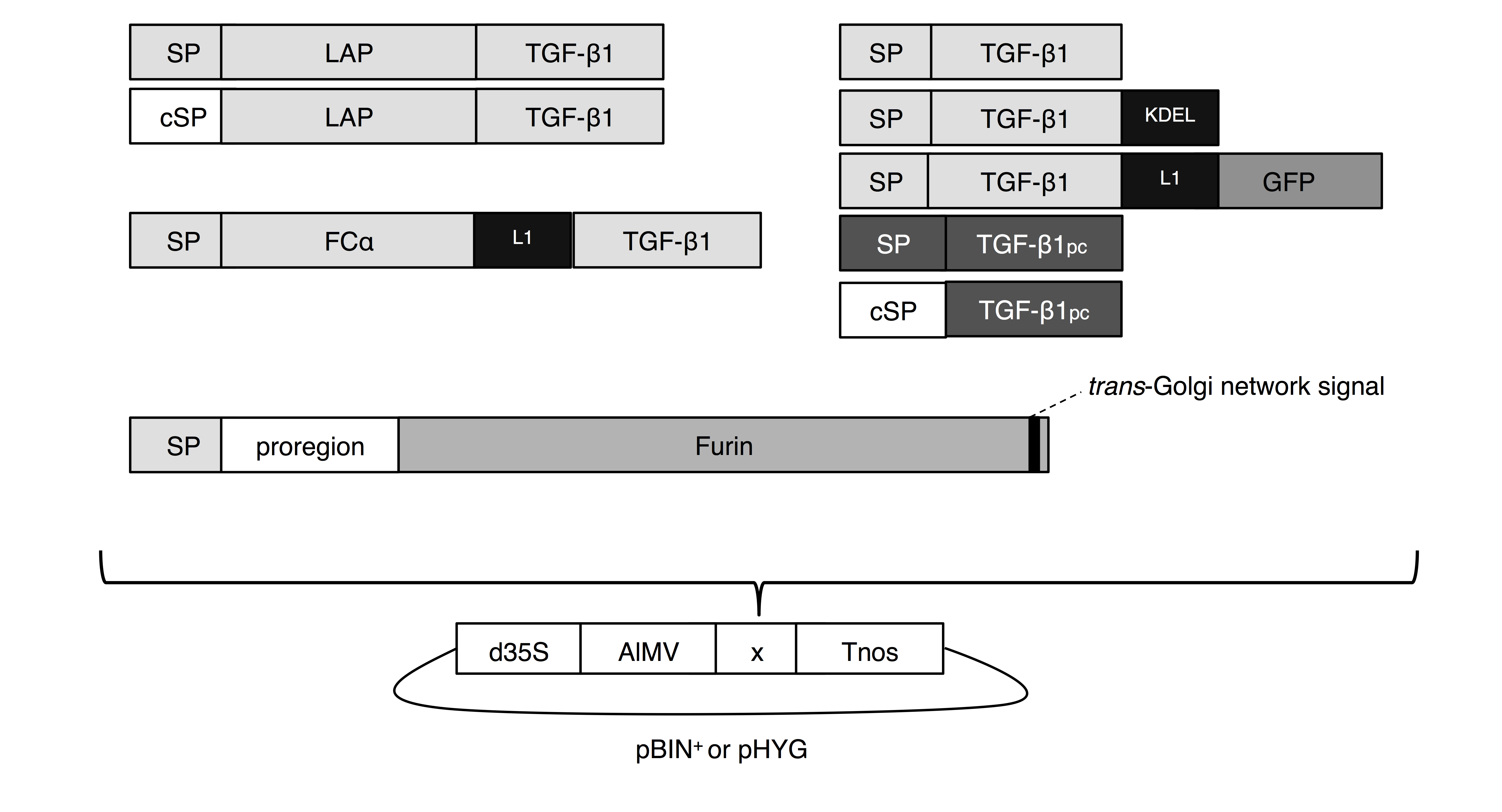

Supplement: Supplementary file 2 — Figure S2 A schematic overview of all the constructs used in this study. [file PBI-14-1695-s002.tiff]
